# Dynamic and Static Amplitude of Low-Frequency Fluctuation Is a Potential Biomarker for Predicting Prognosis of Degenerative Cervical Myelopathy Patients: A Preliminary Resting-State fMRI Study

**DOI:** 10.3389/fneur.2022.829714

**Published:** 2022-04-04

**Authors:** Ningjian Fan, Bing Zhao, LiYun Liu, WeiZhen Yang, Xian Chen, ZhanBin Lu

**Affiliations:** Department of Spinal Surgery, The Second Hospital of Tangshan, Tangshan, China

**Keywords:** functional magnetic resonance imaging, degenerative cervical myelopathy, dynamic amplitude of low-frequency fluctuation, multivariate analysis, Support Vector Machine (SVM)

## Abstract

**Objective:**

The aim of this study was to explore the clinical value of the static amplitude of low-frequency fluctuation (sALFF) and dynamic amplitude of low-frequency fluctuation (dALFF) in the identification of brain functional alterations in degenerative cervical myelopathy (DCM) patients.

**Methods:**

Voxel-wise sALFF and dALFF of 47 DCM patients and 44 healthy controls were calculated using resting-state fMRI data, and an intergroup comparison was performed. The mean of sALFF or dALFF data were extracted within the resultant clusters and the correlation analysis of these data with the clinical measures was performed. Furthermore, whole-brain-wise and region-wise multivariate pattern analyses (MVPAs) were performed to classify DCM patients and healthy controls. sALFF and dALFF were used to predict the prognosis of DCM patients.

**Results:**

The findings showed that (1) DCM patients exhibited higher sALFF within the left thalamus and putamen compared with that of the healthy controls. DCM patients also exhibited lower dALFF within bilateral postcentral gyrus compared with the healthy controls; (2) No significant correlations were observed between brain alterations and clinical measures through univariate correlation analysis; (3) sALFF (91%) and dALFF (95%) exhibited high accuracy in classifying the DCM patients and healthy controls; (4) Region-wise MVPA further revealed brain regions in which functional patterns were associated with prognosis in DCM patients. These regions were mainly located at the frontal lobe and temporal lobe.

**Conclusion:**

In summary, sALFF and dALFF can be used to accurately reveal brain functional alterations in DCM patients. Furthermore, the multivariate approach is a more sensitive method in exploring neuropathology and establishing a prognostic biomarker for DCM compared with the conventional univariate method.

## Introduction

Degenerative cervical myelopathy (DCM) is characterized by acquired stenosis in the cervical spine (1). The prevalence of DCM has been increasing over the years and is currently the most commonly reported nontraumatic spinal cord dysfunction (2). Timely diagnosis and surgical intervention are required to relieve neurological symptoms of DCM (3, 4). Currently, the primary treatment for DCM is decompression surgery. The major surgical indications for DCM include a definite diagnosis of myelopathy (diagnosis based on both clinical symptoms and MR findings) and progressive myelopathy (5). Although these indications are concise and easy to perform, it is challenging for surgeons to decide whether surgical treatment is required for DCM patients with chronic myelopathy lasting for years (to explore whether surgery is no longer effective for these patients) (6). Therefore, simple, accurate prognostic biomarkers for DCM are needed to determine whether patients will be benefited from the surgery.

Researchers previously used images acquired from conventional MRI of the cervical spine to predict neurological recovery of the DCM patients (such as high signal intensity on T2-weighted MR). However, its utility is controversial because information obtained from the spinal cord area is limited (only a small cross-sectional area is accessible) (7, 8). Resting-state fMRI was recently used to establish a prognostic biomarker for DCM patients (9–11). The functional connectivity (FC) between the occipital lobe and the frontal lobe is highly associated with neurological recovery in DCM patients (9). Moreover, the amplitude of low-frequency fluctuation (ALFF) of the frontal lobe has high potential application in predicting the prognosis of DCM patients (10). These results provide preliminary evidence that rs-fMRI can be used for prognostic prediction in DCM patients. However, these studies have some limitations. First, (1) previous studies used FC and ALFF as prognostic biomarkers for DCM patients. These metrics statistically measured the cortical function during the entire scan, and ignored the time-varying characteristics of intrinsic brain activity over time in DCM patients. On the contrary, dynamic ALFF (dALFF) can reflect temporal variability of intrinsic brain activity (12) and shows promise in identifying the pathology of diseases and potential application in the development of prognostic biomarkers. Second, (2) previous studies only performed univariate analyses (such as voxel-wise or region-wise correlation analyses between rs-fMRI metrics and prognosis of DCM patients). Association between brain metrics and clinical variables can be revealed and easily interpreted through univariate analysis. However, the univariate analysis only considers linear relation between the amplitude of a given voxel or connection and clinical measures. The pattern information which is constituted by the clustered voxels or numerous connections among a given network, are therefore ignored in univariate analysis (13–15).

To bridge these gaps, the clinical value of dALFF in the diagnosis of DCM and as a potential prognostic biomarker was explored through MVPA in DCM patients. ALFF and dynamic ALFF were calculated in this study. Mass univariate analysis and multivariate pattern analysis (SVM classification) were performed to explore the differences between DCM patients and healthy controls. Moreover, univariate correlation analysis and MVPA (through support vector regression, SVR) were performed to predict the prognosis of DCM patients following spinal cord decompression surgery. The aim of this study was to explore whether dALFF provides additional information in determining DCM-related pathology, and can serve as an effective prognostic biomarker for DCM patients compared with ALFF.

## Materials and Methods

### Subjects

The local Institutional Review Board approved this cross-sectional study. All the participants provided written informed consent before each procedure. A total of 47 DCM patients and 44 healthy participants matched for age/gender were included in this study (Table 1). Inclusion criteria of DCM patients in the present study were as follows: (1) clear signs and symptoms of spinal cord myelopathy (such as sensorimotor deficits, bowel or bladder dysfunction); (2) MR findings corresponding with the clinical signs and symptoms; (3) patients with no other complications such as heart disease, hepatic disease, and renal disease; (4) Patients with no history of alcohol and drug abuse; (5) patients with no psychological or neurological diseases; (6) patients willing to undergo decompression surgery; (7) patients able to complete the fMRI scan. Furthermore, healthy subjects were recruited to the study through advertisements. Healthy participants with no evidence of spinal cord compression, no other complications, no psychological or neurological diseases, no history of alcohol and drug abuse and ability to complete an fMRI scan were included in this study.

**Table 1:** Demographic data.

	**DCM**	**HC**	***P***
Age	51.3 ± 2.8	51.7 ± 3.6	0.81
Gender (Female)	22 (22)	20 (18)	0.94
Education years	12 ± 2.4	11.7 ± 3.6	0.74
JOA score	11.3 ± 3.2		
JOA recovery rate	70% ± 15.6%		
Disease duration (Month)	30.5 ± 17.4		

*DCM, Degenerative Cervical Myelopathy; HC, Healthy Controls; JOA, Japanese Orthopedic Association*.

### Clinical Evaluation

All DCM patients were first evaluated by a senior orthopedic surgeon based on the Japanese Orthopedic Association (JOA) score (16) for assessing preoperative severity of sensorimotor symptoms (denoted as preoperative JOA score) immediately before fMRI scan. Further, the JOA score was used by the same surgeon postoperatively for evaluating patients 6 months after decompression surgery to obtain the postoperative JOA score for each patient. The JOA recovery rate was calculated to reveal the recovery for DCM patients following surgery. The JOA recovery rate (17) as shown below:


$$\begin{array}{l} JOA\;recovery\;rate=\\ \frac{\left(Postoperative\;JOA\;scores-Preoperative\;JOA\;scores\right)}{\left(17-Preoperative\;JOA\;scores\right)} \end{array}$$

Moreover, several patient characteristics related to the prognosis of the DCM patients were evaluated in this study including age, smoking status, preoperative neurological function (defined as preoperative JOA score), and gait disturbance.

### fMRI Data Acquisition and Preprocessing

Functional MRI data were obtained using a 3.0 T MR scanner (Discovery MR750, General Electric) with a 20-channel phased-array head coil. All participants were instructed to keep their mind clear and focused on the cross displayed on the screen. Further, participants were required to avoid specific and strong ideological activities during the entire scan period. Sponge pads were packed around the heads of participants to minimize unconscious head movement. Earplugs were placed inside the participants' ears to minimize noise. fMRI data were collected using a gradient Echo-Planar pulsed Imaging (EPI) sequence with the following parameters: 180 time points; repetition time = 2,000 ms; echo time = 30 ms; flip angle = 80°; the field-of-view = 240 × 240 mm; matrix = 64 × 64; the number of slices = 48 slices; and slice thickness = 3.0 mm. T1 structure images were obtained using three-dimensional T1-weighted image (3D T1WI) for coregistration and normalization of functional images with the following parameters: voxel size = 1.0 × 1.0 × 1.2 mm^3^; flip angle = 13°; echo time (TE) = 3.0 ms; repetition time (TR) = 7.8 ms; 180 sagittal slices; within plane field-of-view = 256 × 256 mm^2^ and slice thickness = 1.0 mm.

Functional MRI data were preprocessed using Data Processing Assistant for resting-state fMRI (DPARSF; http://www.restfmri.net/forum/DPARSF) based on Statistical Parametric Mapping 12 (SPM 12) platform. The first 10 volumes from each functional scan were removed to ensure acclimatization to the scanning environment and magnetization stabilization. The remaining 170 images were then motion corrected to remove timing differences between slices and head movement. Subsequently, fMRI data with motion displacement of more than 1.5 mm (in any of the *x, y*, or *z* directions) or 1.5° rotation of angular motion were excluded from further analysis (3 DCM patients were excluded). The mean rs-fMRI images were then coregistered to the structural image, spatially normalized to the Montreal Neurological Institute (MNI) standard space, and resampled into 3 × 3 × 3 mm cubic voxel. Nuisance covariates, including 24 head motion parameters and signals of white matter and CSF, were regressed to minimize non-neural signals. In this step, the global signal was not regressed out due to that the regression of the global signal is still controversial because it can introduce artifactual negative correlations (18–21). Therefore, the global signal was not regressed in our current study. In addition, a scrubbing step for high-motion timepoints was performed. The threshold for scrubbing was set at 0.5 as calculated by the FD Jenkinson method. Images exceeding this threshold were scrubbed using the cubic spine method (scrubbing timepoints before bad timepoints: 2; scrubbing time-points after bad timepoint). Notably, 3 patients and 2 healthy controls had one timepoint that exceeded the threshold and 2 patients and 2 healthy controls had two timepoints that exceeded the threshold. Finally, rs-fMRI images were filtered within 0.01–0.08 Hz and spatially smoothed with a Gaussian kernel of 6 × 6 × 6 mm^3^ full width at half maximum (FWHM).

### Amplitude of Low-Frequency Fluctuation and dALFF Variance Calculation

Amplitude of low-frequency fluctuation was calculated using the DPARSFA MATLAB toolbox. Detailed calculation procedures were as follows: (1) fast Fournier transformation was performed for the time series of each voxel to change the time domain to frequency domain; (2) the voxel-wise square root of the power spectrum was computed and averaged across 0.01–0.08 Hz; (3) the resultant averaged square root represented the ALFF of each voxel; (4) the ALFF was then Z-scored.

Dynamic ALFF was calculated using the Dynamic BC toolbox. A sliding-window approach was utilized to reflect the temporal variability of intrinsic brain activity. The window length was considered as an important parameter that determined the robustness of the results. Previous studies report that an appropriate window length ranges from 40 to 100 s (12, 22, 23). Therefore, in this study, 22 TR (44 s) was chosen as the window size, and 1 TR as the window step (23). Furthermore, the 50 TR (100 s) and 36 TR (72 s; the average of 22 TR and 50 TR) were also chosen as the window size to further validate our results obtained from 22TR. ALFF was then calculated using as described earlier. The ALFF map for each sliding window was then obtained, as well as the dALFF variance which indicates the temporal stability of intrinsic neural fluctuations.

### Univariate Analysis

Standard rs-fMRI analysis was performed using mass univariate analysis to explore the functional differences (such as ALFF and dALFF) between DCM patients and healthy controls. Voxel-wise two-sample *t*-test was performed within the gray matter using age, gender, and education years as covariates. The significance threshold was set at *P* ≤ 0.001 and was corrected for multiple comparisons with family-wise error (FWE) correction at the cluster level using SPM12 (http://www.fil.ion.ucl.ac.uk/spm). The corresponding corrected *P*-value was ≤ 0.05,

The resultant clusters, which exhibited significant between-group differences, were identified. Mean zALFF within these clusters were extracted and the relationship with clinical measures (including preoperative JOA scores and JOA recovery rate) was explored through correlation analysis.

### Multivariate Pattern Analysis

Multivariate pattern analysis (MVPA) uses a pattern classifier to identify pattern differences for neural activities between different conditions or between patients and healthy participants. Only the amplitude of a single-voxel or a single the region of interest (ROI) was considered at a time in univariate analysis. Notably, neural activity information is represented by the amplitude of the neural signal and exists in a pattern composed of multiple voxels. On the contrary, MVPA considered the spatial pattern of neural signals across the whole brain or across voxels within a predefined area.

In this study, MVPA analysis was performed using the MVPANI toolbox (24) (http://funi.tmu.edu.cn) and LibSVM's implementation of linear Support Vector Machine (SVM) using default parameters. The linear kernel was used and the penalty coefficient (c) was set to 1. Other parameters were not adjustable therefore the default parameters were used.

### Multivariate Pattern Analyses: DCM Patients vs. Healthy Controls

#### ROI-Wise MVPA

Due to the relatively small sample size in our study, a whole brain gray matter mask inevitably led to the overfitting of the model. Therefore, a sensorimotor mask was acquired from Shirer et al. (25) (the spatial distribution of the mask was shown in Supplementary Figure 1). MVPA was then applied within the sensorimotor cortices given that previous literature reports of cortical alterations of DCM were mainly located at the sensorimotor network (9, 26–29).

A leave-one-out-cross-validation (LOOCV) procedure was conducted to validate our classification model owing to the relatively small sample size in this study. Detailed procedures for performing LOOCV in this study were as follows: (1) one sample (ALFF or dALFF map) from the dataset was held out as the testing data, and the rest of the dataset were used for training the classification model; (2) the trained model was then tested using the held-out sample (held-out sample from step 1); (3) a binary classification accuracy was obtained for this data point (100% for right classification, 0% for the wrong classification); (4) subsequently, step 1 to step 3 were repeated until all the samples were held-out once as the testing data; (5) averages of the classification accuracies of all folds were obtained (the number of correct classifies divided by the number of samples).

Sensitivity maps for each fold were generated. In these maps, the value of each voxel represented its weight in the SVM model. A high weight for a given voxel indicated that the voxel had a strong contribution to the classifier model in predicting whether the participant is a DCM patient. The sign of the model weight denoted the preference for DCM-related neural activities or healthy conditions being compared in classification. This implies that when performing the classification “DCM patients vs. healthy controls”, a positive sign indicated that this voxel exhibited a higher ALFF (or dALFF) in DCM patients, whereas a negative weight implied that this voxel exhibited a higher ALFF (or dALFF) in healthy participants. In this study, voxels whose signs were consistent across all folds were explored further. This indicates that voxels consistently showing positive (or negative) weight across all folds were presented in this study.

Null distributions of the classification accuracies were obtained using a permutation test to explore whether the classification accuracy was above the chance level (50%). Samples were randomly labeled and thus no information on the grouping of the DCM patients and healthy controls was provided when training the classifiers. The procedures were repeated 1,000 times for each classification and a nonparametric *P*-value was then obtained for each classification (the proportion of the null distribution that is equal to or higher than the actual classification accuracy).

#### Region-Wise MVPA

In region-wise MVPA, LOOCV procedures similar to those used for whole-brain-wise MVPA were performed for each brain region defined by both AAL atlas and Brainetome atlas (30). A brief description of the region-wise MVPA analysis is as follows: First, the classification accuracies of each brain region were obtained by the LOOCV approach. The null distribution of the classification accuracy for each brain region was then obtained by permutation analysis (repeated for 1,000 times). The maximum classification accuracy across all brain regions was obtained for each permutation step. Subsequently, a null distribution comprising all maximum classification accuracies across brain regions was obtained (in a total of 1,000 accuracies). *P*-values for all classification accuracies were calculated using null distribution (comprising all maximum classification accuracies across brain regions). *P*-values were automatically corrected by the FEW method for multiple comparison analysis (31). The significance level was set to *P* < 0.05 after FWE correction for multiple comparisons. ALFF and dALFF were used as features for region-wise MVPA. The rationale for this analysis was to give a more detailed spatial distribution (complement for the sensitivity map) for brain regions associated with DCM.

### Multivariate Pattern Analyses: Prognosis Prediction for DCM Patients

#### ROI-Wise MVPA

In this analysis, we also restrict our analysis within sensorimotor cortices using the previously illustrated templates (Supplementary Figure 1). LOOCV was also performed for validation of our prediction model.

The detailed LOOCV procedures were as follows: (1) one data point (such as 1 fold) within the dataset was held-out as the testing data; (2) SVR model was then trained using the other portion of the dataset; (3) the trained model was then tested based on the held-out data (testing data); (4) a prediction value was then acquired along with the error between the predicted label and the true label; (5) step 1–step 4 were repeated until all data points were held-out once as the testing data; (6) finally, correlation coefficient and the root mean square error (RMSE) between the predicted labels and the true labels were obtained.

#### Region-Wise MVPA

Leave-one-out-cross-validation procedures similar to those used for ROI-wise MVPA were performed in region-wise MVPA for each brain region defined by both AAL atlas and Brainetome atlas. The region-wise MVPA analysis was conducted as follows: first, correlation coefficients of each brain region were obtained by the LOOCV approach. The null distribution of the correlation coefficient for each brain region was then obtained by permutation analysis. The maximum correlation coefficient across all brain regions was obtained for each permutation step. Subsequently, a null distribution comprising all maximum correlation coefficients across brain regions was obtained. *P*-values for all correlation coefficients were calculated using the null distribution approach (comprising all maximum correlation coefficients across brain regions). The *P*-values were automatically corrected by the FEW method for multiple comparison correction (31). The significance level was set to *P* < 0.05 after FWE correction. ALFF and dALFF were used as features for region-wise MVPA. The aim of this analysis was to give more detailed spatial distributions (complement the sensitivity map) for brain regions associated with the prognosis of DCM patients.

## Results

### Degenerative Cervical Myelopathy Patients Exhibited Lower dALFF and Higher sALFF Compared With Healthy Controls

**Figure 1:**
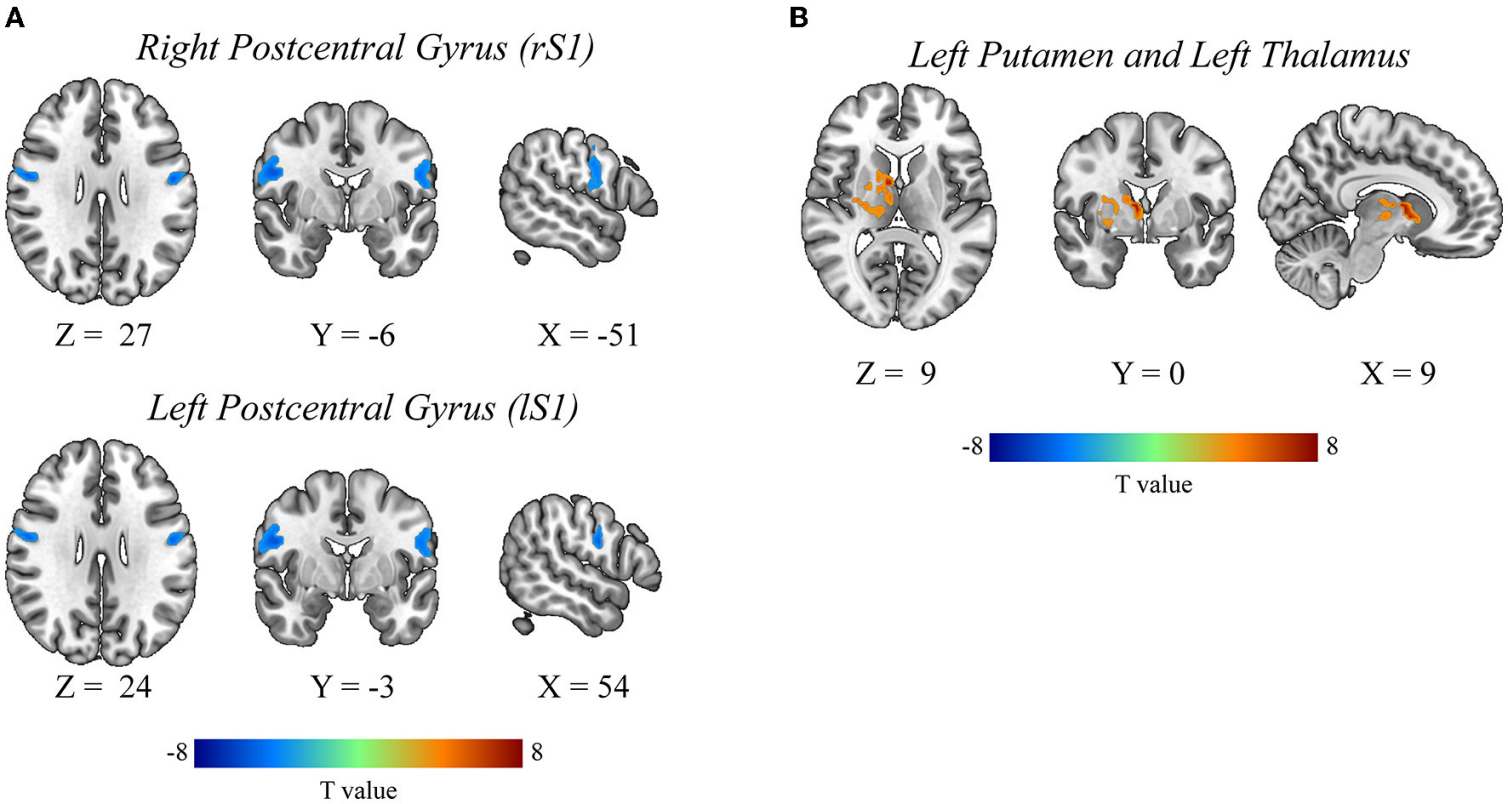
The univariate differences in dALFF and sALFF between DCM patients and healthy controls. DCM, Degenerative Cervical Myelopathy; HC, Healthy Controls; sALFF, static amplitude of low-frequency fluctuation; dALFF, dynamic amplitude of low-frequency fluctuation. **(A)** The dALFF differences between DCM and HC. **(B)** The sALFF differences between DCM and HC.

Degenerative cervical myelopathy exhibited significantly lower dALFF within bilateral postcentral gyrus (S1), and exhibited significantly higher sALFF within left putamen and left thalamus relative to those of healthy participants (Figure 1). Details of each cluster are presented in Table 2. Our results from different window sizes were also consistent with our main result. The details of each cluster can be found in Supplementary Figure 2 and Supplementary Table 1.

**Table 2:** The dALFF and sALFF differences between DCM patients and healthy controls.

**Brain region**	**MNI coordinates**			**T value**	**Voxel size**
**dALFF**					
lS1	−51	−6	27	−5.7	97
rS1	54	−3	24	−5.4	55
sALFF					
lPutamen, lTHA	9	0	9	8.1	196

*dALFF, dynamic amplitude of low-frequency fluctuation; sALFF, static amplitude of low-frequency fluctuation; DCM, degenerative cervical myelopathy; lS1, left postcentral gyrus; rS1, right postcentral gyrus; lTHA, left thalamus*.

### Brain Variables Were Not Correlated With Clinical Characteristics as Exhibited by Univariate Analysis

In this study, brain variables were not significantly correlated with clinical measures within brain regions as shown by univariate analysis (Table 3, all *p* > 0.05). Furthermore, the dALFF acquired from different window sizes were also not correlated with clinical measures.

**Table 3:** The correlation coefficients between altered brain function and clinical measures.

**dALFF**		
	lS1	rS1
JOA score	−0.02	0.02
JOA recovery rate	−0.19	−0.17
**sALFF**		
	lTha	
JOA score	0.10	
JOA recovery rate	0.26	

*The correlation coefficients between dALFF/sALFF and clinical measures. JOA, Japanese Orthopedic Association. All p > 0.05*.

### Multivariate Pattern Analysis Shows Differences in dALFF and sALFF in DCM Compared With Healthy Controls

**Figure 2:**
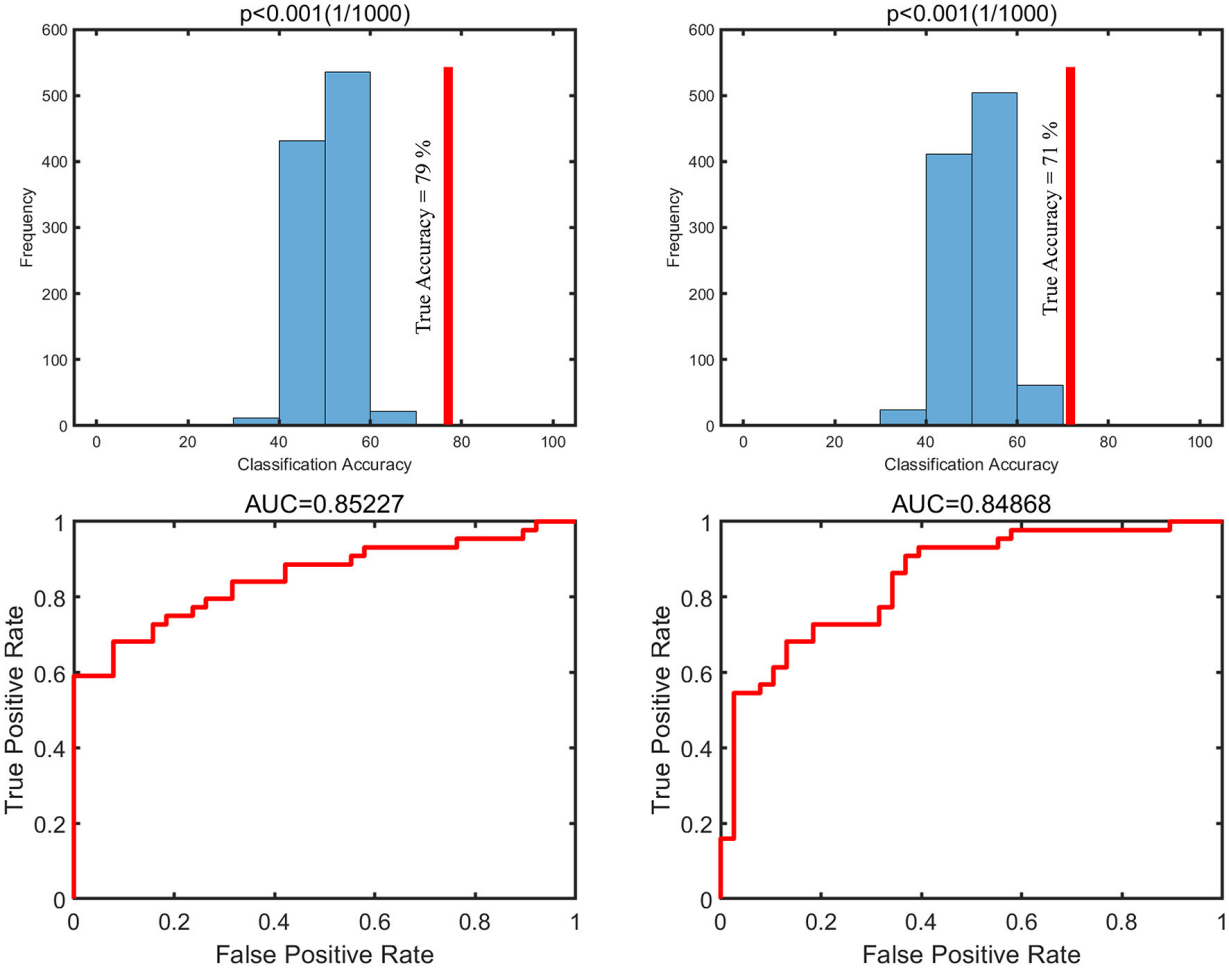
The null distributions of the classifications and receiver operator characteristic curve (ROC curve) for DCM vs. HC using sALFF and dALFF. DCM, Degenerative Cervical Myelopathy; HC, Healthy Controls; sALFF, static amplitude of low-frequency fluctuation; dALFF, dynamic amplitude of low-frequency fluctuation; AUC, area under the curve.

Further MVPA analysis revealed differences in dALFF and sALFF between the two groups which were not exhibited by univariate analysis. MVPA analysis was performed to explore whether the sensorimotor network functional pattern of dALFF or sALFF can be used to distinguish DCM patients from the healthy controls. The results showed that DCM patients could be successfully identified from healthy controls using both sALFF and dALFF as features with a significantly high-classification accuracy (71% for sALFF, 79% for dALFF) (Figure 2).

**Figure 3:**
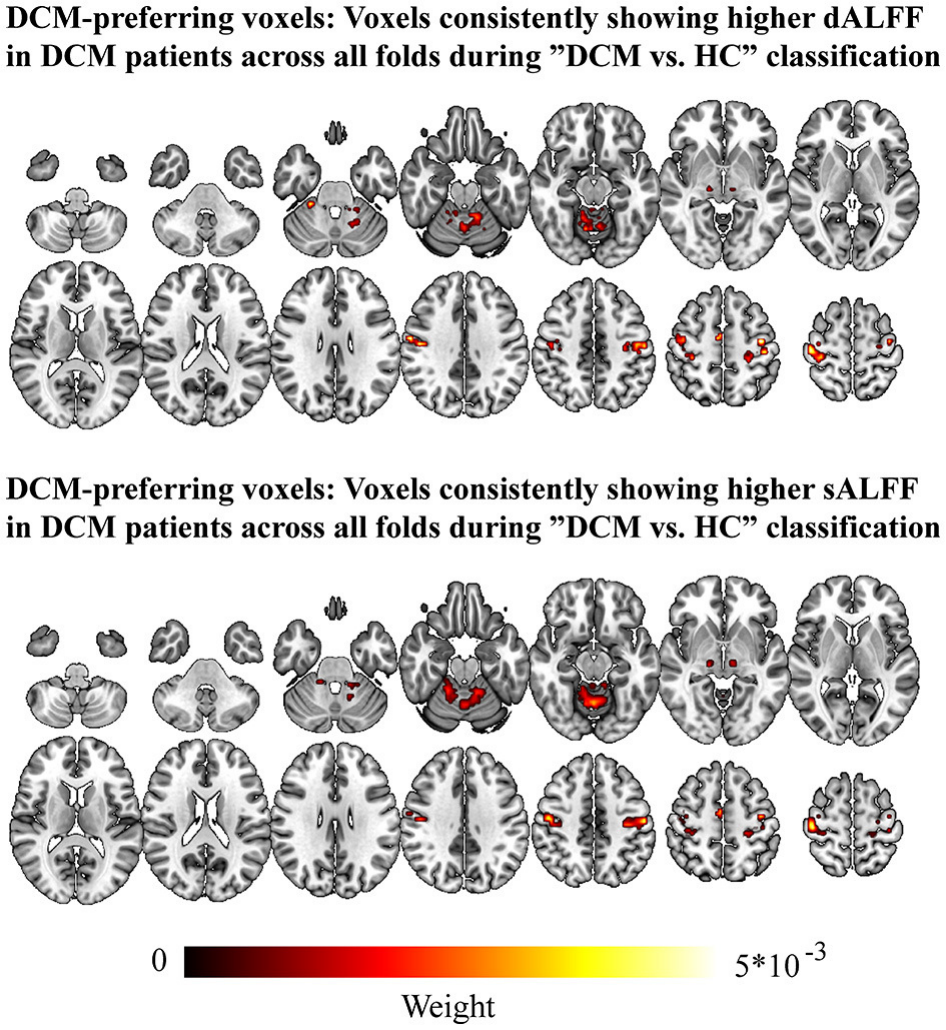
Voxels showed higher weight for classification for “DCM vs. HC” across all folds. DCM, Degenerative Cervical Myelopathy; HC, Healthy Controls.

Sensitivity maps for classifications (DCM vs. HC) are presented in Figure 3. Voxels exhibited high weights (consistently positive across all folds). These voxels were mainly located at the sensorimotor cortices (bilateral SMA, M1, S1, posterior cerebellum). These results were also validated by our results from dALFF analysis using different window sizes (Supplementary Figure 4).

**Figure 4:**
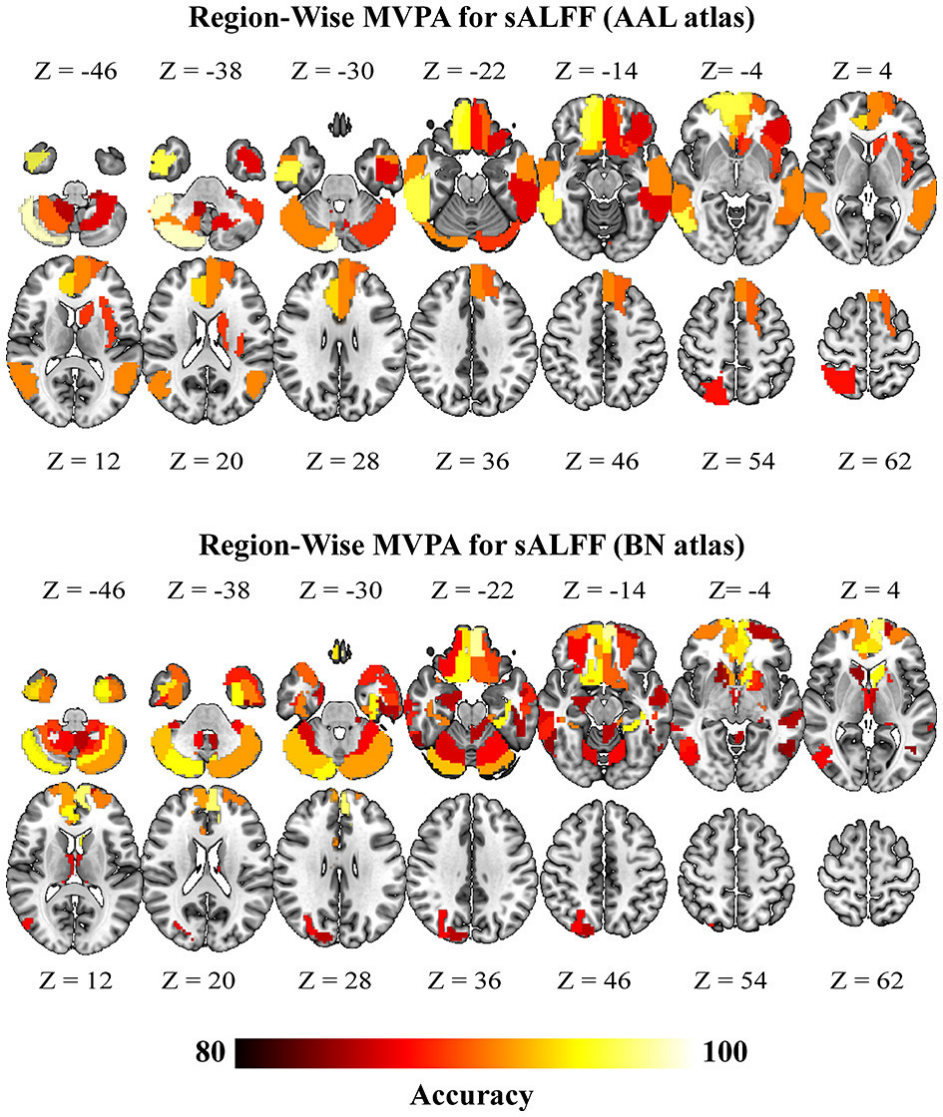
Brain regions with significant classification accuracy for “DCM vs. HC” using the static amplitude of low-frequency fluctuation (sALFF) (*p* < 0.05, FWE for multiple comparison correction). DCM, Degenerative Cervical Myelopathy; HC, Healthy Controls; AAL, Anatomical Automatic Labeling; BN, Brainnetome.

**Figure 5:**
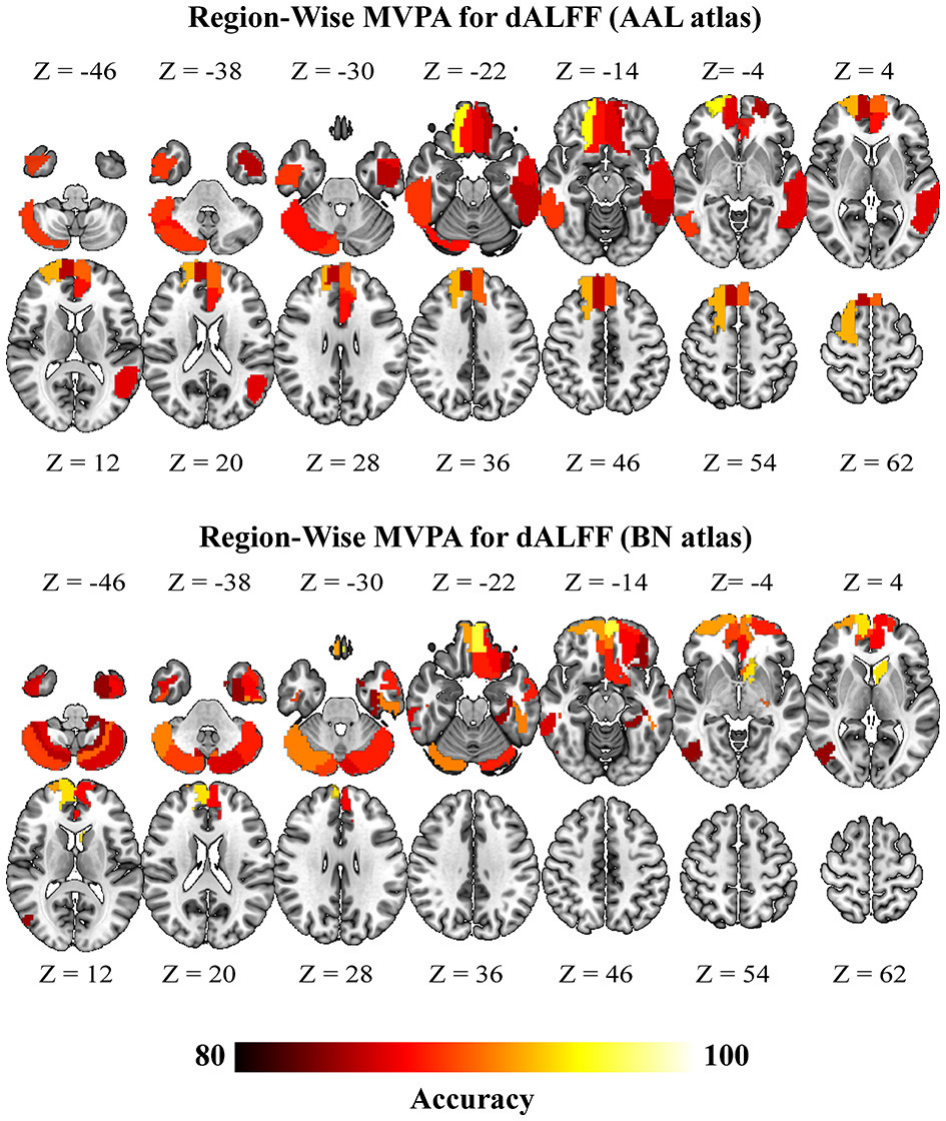
Brain regions with significant classification accuracy for “DCM vs. HC” using the dynamic amplitude of low-frequency fluctuation (dALFF) (*p* < 0.05, FWE for multiple comparison correction). DCM, Degenerative Cervical Myelopathy; HC, Healthy Controls; AAL, Anatomical Automatic Labeling; BN, Brainnetome.

Region-wise MVPA further revealed that brain regions exhibited higher classification. Notably, sALFF of the brain regions that exhibited significant accuracies were mainly located at the bilateral frontal cortices and bilateral temporal gyri (Figure 4) (consistent between two different atlases; brain regions highlighted in Supplementary Figure 2). The brain regions that exhibited significant accuracies for dALFF were also located at the bilateral frontal cortices, bilateral inferior temporal gyrus, bilateral inferior occipital gyrus, and left posterior cerebellum (Figure 5) (consistent between two different atlas, brain regions highlighted in Supplementary Figure 3). Our results using different window sizes for dALFF were consistent with our main results that the brain regions exhibited higher accuracies were located at the frontal cortices and sensorimotor cortices (details can be found in brain regions highlighted in Supplementary Figures 4, 5).

### Multivariate Pattern Analysis: Prognosis Prediction for DCM Patients

**Figure 6:**
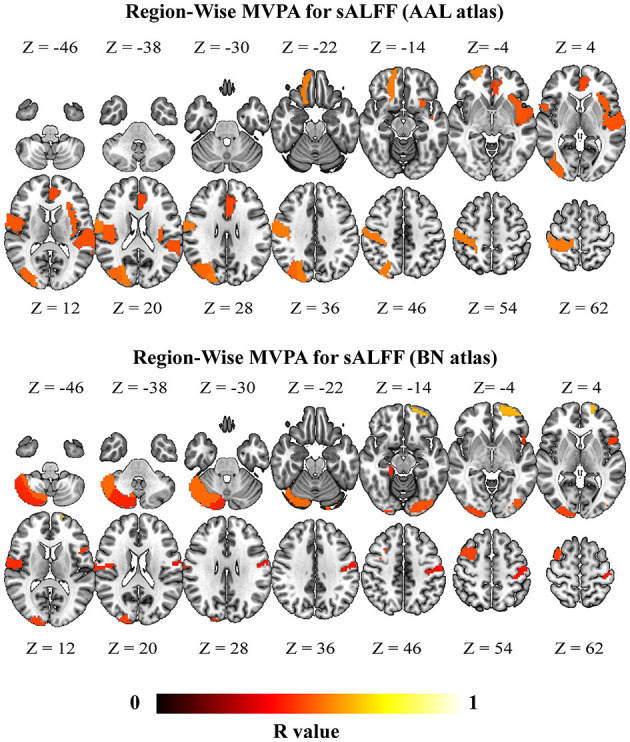
Brain regions with significant correlation coefficients in the prediction of JOA recovery rate using the static amplitude of low-frequency fluctuation (sALFF) (*p* < 0.05, FWE for multiple comparison correction). DCM, Degenerative Cervical Myelopathy; HC, Healthy Controls; AAL, Anatomical Automatic Labeling; BN, Brainnetome.

**Figure 7:**
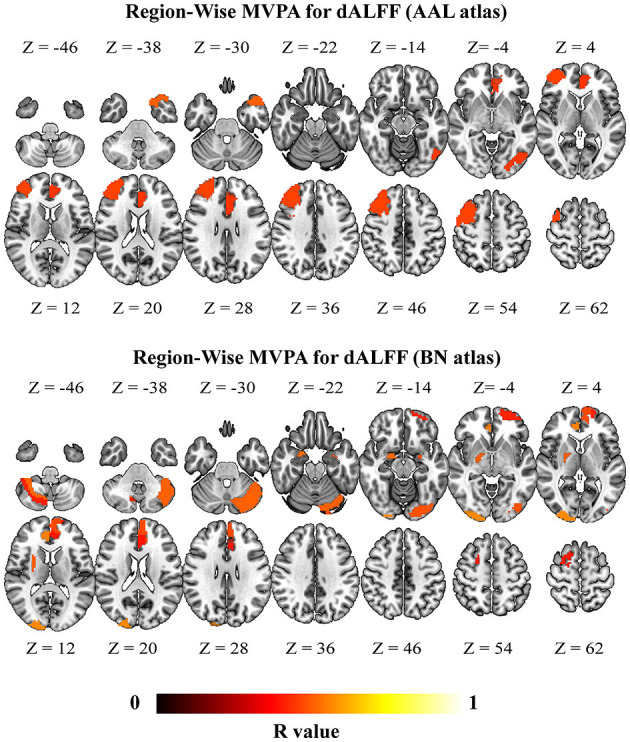
Brain regions with significant correlation coefficients in the prediction of JOA recovery rate using the dynamic amplitude of low-frequency fluctuation (dALFF) (*p* < 0.05, FWE for multiple comparison correction). DCM, Degenerative Cervical Myelopathy; HC, Healthy Controls; AAL, Anatomical Automatic Labeling; BN, Brainnetome.

Findings from region-wise MVPA prediction for JOA recovery showed that the brain regions that successful prediction was mainly located at the frontal cortices for sALFF (Figure 6) (consistent between two different atlas, brain regions highlighted in Supplementary Figure 6). In addition, brain regions that exhibited significant correlations between predicted labels and actual labels for dALFF were mainly located at the frontal cortices, left insular and posterior lobe of the cerebellum (consistent between two different atlas, brain regions highlighted in Supplementary Figure 7). (Figure 7) Our results using different window sizes for dALFF were consistent with our main results that the brain regions that exhibited successful prediction was located at the frontal cortices, left insular and posterior lobe of the cerebellum (details can be found in brain regions highlighted in Supplementary Figures 8, 9).

## Discussion

The main findings for this study were as follows: (1) the results from the univariate analysis revealed brain functional alterations in the DCM patients. However, these alterations were not correlated with clinical symptoms. (2) MVPA successfully classified DCM patients and distinguished them from healthy adults using sALFF and dALFF as features. (3) MVPA showed potential application in predicting the prognosis of DCM patients.

### Degenerative Cervical Myelopathy Patients Exhibited Increased sALFF Within Putamen and Thalamus, and Decreased dALFF Within the Bilateral S1

Thalamus is the first region for transmitting sensory information from the spinal cord, and plays an important role in the neuropathology of DCM (32, 33). Zhou et al. reported that DCM patients exhibited increased FC between the thalamus and superior frontal gyrus, between thalamus and precentral gyrus (M1) in slow-4 frequency band (0.027–0.073 Hz) relative to that of healthy adults. A study conducted full-band (0.01–0.08 Hz) FC analysis and reported increased FC between the thalamus and bilateral lingual gyrus/cuneus (33). Moreover, DCM patients manifested decreased FC between the right thalamus and bilateral paracentral lobe/precentral gyrus following decompression surgery but exhibited significantly increased FC between right thalamus and pons/superior temporal gyrus compared with that of healthy controls (32). These results represent compensatory changes following long-term compression of the spinal cord. The functional changes indicate that sensorimotor cortices (such as M1) recruit the adjacent cortex to compensate for the functional deficits of myelopathy (34–36). In summary, the observed increased sALFF within the thalamus in this study is attributed to response to myelopathy, and cortical reorganization was initiated to compensate for the functional deficits in the DCM patients. The postcentral gyrus (S1) is a key region implicated in cortical reorganization in DCM pathology. The recent studies report that functional, structural, and metabolic alterations occur within S1 in the DCM patients (37–39). These alterations are considered as the sensory-motor cortical plasticity, which is the dynamic potential of the brain to reorganize following secondary injury during the progression of chronic spinal cord injury. The findings on sALFF and dALFF changes in this study were consistent with results from previous studies, and thus, they are a result of cortical reorganizations following long-term myelopathy. Notably, no significant correlation between brain alterations and clinical measures was observed in the DCM patients. There are two possibilities for this phenomenon, first only used JOA score was used for determining disease DCM severity which only considered the patient's signs and symptoms and ignored the structural changes of the spinal cord. Therefore, the JOA score may not be a comprehensive indicator for evaluating the severity of DCM. Second, in this section, only univariate correlation analysis was performed, and the nonlinear association between brain alterations and clinical measures may have been ignored.

### Research Imaging Institute-Wise Functional Pattern of Both sALFF and dALFF Successfully Distinguished DCM Patients From Healthy Controls

Cortical functional alterations of the brain have been identified in the past decades. Widespread brain regions including sensorimotor cortices (such as M1, S1, SMA) (37–39), occipital cortices (such as primary visual cortices, secondary visual cortices) (11, 40, 41), frontal cortices (such as superior and middle parts) (10, 38, 42), default mode network (such as medial frontal cortices, posterior cingulate cortices, angular gyri) (29), temporal cortices (including inferior part and superior part) (29), and cerebellum (including cerebellum crus, posterior cerebellum) are implicated in DCM pathogenesis (40, 41). However, these studies only conducted univariate analysis (such as voxel-wise independent *T*-test) to explore group differences between DCM patients and healthy controls. Although positive results have been obtained, this approach only detects the amplitude differences in brain alterations within a single voxel, and ignores the pattern information attributed to multiple voxels or brain regions. Therefore, the multivariate approach gives a more comprehensive description of the functional alteration pattern of the brain. It is important to test whether the sensorimotor cortices pattern of these functional metrics (such as sALFF and dALFF) can successfully distinguish the DCM patients from healthy controls before identification of DCM-related brain regions. The findings of this study showed high-classification accuracies (71% for sALFF and 79% for dALFF) in classifying DCM patients and healthy controls. This result indicated that the functional pattern of the sensorimotor cortices function is a potential indicator for classifying DCM patients and healthy controls. Initially, this did not appear to have clinical significance, as the diagnosis of DCM currently only included the clinical symptoms of myelopathy and corresponding MR findings. However, for the DCM patients with light symptoms and ambiguous compression on cervical MR, it is challenging for clinicians to determine whether these patients have spinal cord compression and whether these symptoms are progressive (1, 4, 6, 43, 44). The results of this study provide preliminary evidence that DCM-related information is correlated to the functional patterns of the brain and can be used for the diagnosis of DCM. Further studies should explore the utility of sALFF or dALFF for the diagnosis of patients with light symptoms and for predicting the progression of DCM.

### Several Brain Regions Played a Role in the Classification of DCM Patients and Healthy Controls

A multivariate approach was employed to classify DCM patients and healthy controls based on sensorimotor cortices sALFF and dALFF as classification features. The contribution of each voxel to the classifications was determined (the voxels consistently showed a response preference for DCM patients (i.e., voxels with positive weights) in the two classifications). The DCM-preferring voxels were located in the bilateral S1, M1, SMA, and cerebellum vermis. Similar observations were obtained in the regional classification, indicating that the brain regions had significant classification accuracy. Evidence from the past decades has demonstrated that these brain regions are associated with DCM, and this has been confirmed by results from MVPA. Frontal cortices are crucial in motor planning and motor control in human (45–48). Recent studies have also shown that the sALFF in the frontal lobe is a potential biomarker for predicting the prognosis of DCM patients after undergoing decompression surgery (10). However, the regions identified in this study are slightly different from those reported previously. Nevertheless, the present results point to the possibility that frontal cortices may contribute to the pathomechanism of DCM. Moreover, a higher number of brain regions showed good classification accuracy in the region-wise classification for dALFF than for sALFF. To our knowledge, no study has reported whether dALFF can reveal changes in brain regions in the DCM patients. In this study, results showed that dALFF may be a more sensitive metric for revealing functional alterations in the DCM patients. Therefore, dALFF alone or combined with sALFF may more accurately reveal the neural mechanism of DCM and predict the prognosis of DCM patients after undergoing conservative or surgical intervention. We, however, note that the aim of classifying DCM patients and healthy controls was not to distinguish DCM patients from healthy controls. Instead, our aim was to first determine whether rs-fMRI can describe disease-related information through a multivariable model analysis. The successful discrimination of DCM patients and healthy controls provided evidence that the MVPA approach can detect DCM-related information, hence, can be exploited to develop a multivariate prediction model.

### Static ALFF and dALFF Showed the Potential to Predict the Prognosis of DCM Patients After Undergoing Decompression Surgery

Several clinical studies have investigated factors influencing the prognosis of DCM patients after undergoing spinal cord decompression surgery. Identification of such factors will help surgeons to make surgical decisions, hence reducing unnecessary suffering to patients and wastage of medical resources. Several clinical factors including diseases duration, preoperative severity of myelopathy, age, smoking, and the presence of a high signal in cervical T2-weighted images (6, 49, 50) have been found to be associated with the prognosis of DCM. However, they cannot accurately predict the prognosis of DCM patients (1, 6). Therefore, other approaches such as electromyography (8), blood biomarkers (51), PET-CT (52), spinal cord DTI (53) have been employed to identify more effective prognostic markers. It has been shown that these imaging biomarkers can accurately predict the prognosis of DCM. Recent studies used rs-fMRI to identify brain biomarkers that can predict DCM prognosis (9, 10). Results showed that the amplitude of ALFF within the superior frontal gyrus influenced the prognosis of DCM. However, these results were obtained using massive univariate analysis (e.g., univariate correlation analysis) and only considered the amplitude of a given voxel or average signal amplitude within in a given ROI, regardless of the pattern information contained in the multivoxels. To our knowledge, our study is the first to use both static and dynamic ALFF to establish a prognostic biomarker for DCM. Our results demonstrate that static and dynamic ALFF can be exploited to determine prognostic biomarkers for DCM. Furthermore, we found that a higher number of brain regions, which had significant correlation coefficients between the predicted JOA recovery rate and actual JOA recovery rate, were found in region-wise prediction analysis for dALFF than for sALFF. These results indicate that dALFF may be more sensitive than sALFF in identifying brain regions associated with the prognosis of DCM. Overall, this study provides preliminary evidence that resting-state fMRI indicators (e.g., sALFF, dALFF) can be used to predict prognostic outcomes of patients with DCM after undergoing decompression surgery.

## Limitations

This study has several limitations. First, postoperative fMRI data were collected because of the artifacts and heating problems caused by surgical implants. Such data will be collected and analyzed in our future studies. Second, all the patients included underwent long-term conservative intervention before the study. Therefore, future studies should enroll drug-naïve DCM patients. Third, we only analyzed dALFF/sALFF. In the future, other resting-state fMRI metrics such as FC, regional homogeneity (ReHo), and FC strength (FCS) should be analyzed to establish a more accurate prognostic model. Fourth, this study enrolled 47 patients, which is a relatively small sample size. Future large-scale studies are needed to validate these results. At last, the multivariate approach employed in this study could detect a nonlinear association between brain variables and clinical outcomes, however, it could not provide a comprehensible interpretation of these associations. Therefore, other multivariate approaches should be adopted to interpret these associations in future studies.

## Conclusions

In summary, sALFF and dALFF can reveal the functional alterations in brain regions of patients with DCM. Furthermore, a multivariate approach is more sensitive than the conventional method in revealing the neuropathological mechanisms and establishing a prognostic biomarker for DCM.

## Data Availability Statement

The raw data supporting the conclusions of this article will be made available by the authors, without undue reservation.

## Ethics Statement

The studies involving human participants were reviewed and approved by the Second Hospital of Tangshan. The patients/participants provided their written informed consent to participate in this study.

## Author Contributions

ZL designed the study. NF analyzed the data and wrote the manuscript. LL, WY, and XC collected the data. All authors contributed to the article and approved the submitted version.

## Conflict of Interest

The authors declare that the research was conducted in the absence of any commercial or financial relationships that could be construed as a potential conflict of interest.

## Publisher's Note

All claims expressed in this article are solely those of the authors and do not necessarily represent those of their affiliated organizations, or those of the publisher, the editors and the reviewers. Any product that may be evaluated in this article, or claim that may be made by its manufacturer, is not guaranteed or endorsed by the publisher.
