# Supplementary material for: Dynamic and Static Amplitude of Low-Frequency Fluctuation Is a Potential Biomarker for Predicting Prognosis of Degenerative Cervical Myelopathy Patients: A Preliminary Resting-State fMRI Study
Source: Front Neurol. 2022 Apr 4;13:829714. doi: 10.3389/fneur.2022.829714 (PMC9013796; doi:10.3389/fneur.2022.829714)
Supplement: Supplementary file 1 [file Table_1.DOCX]

**Sup-Figure 1**


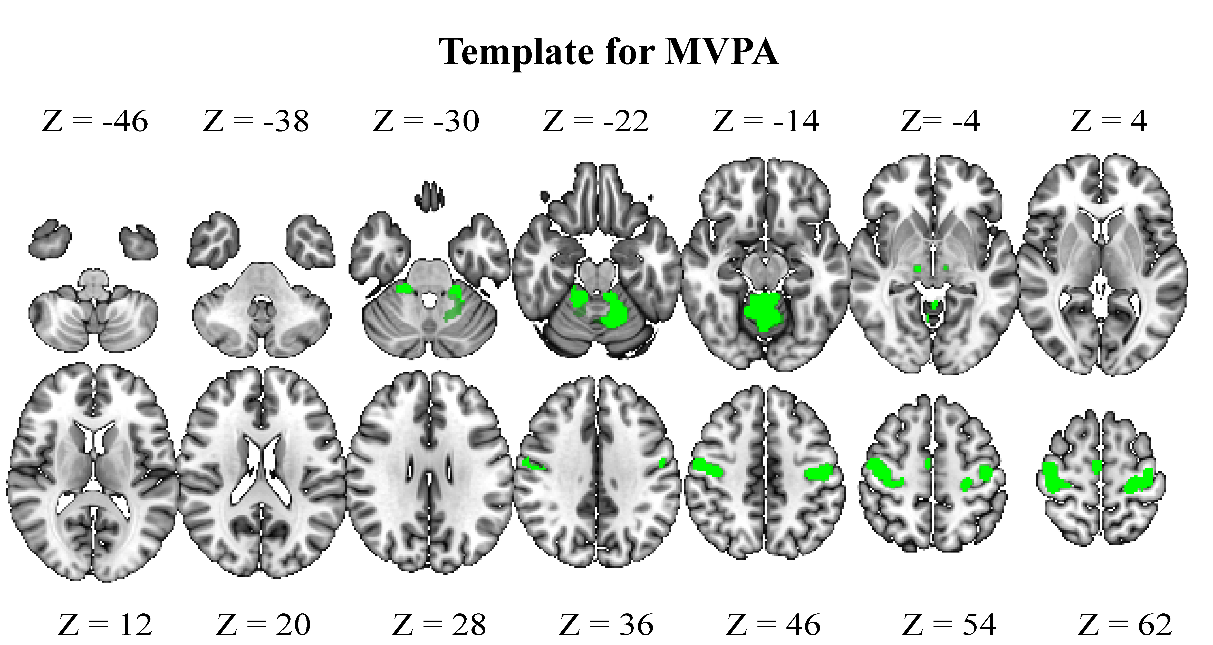


The spatial distribution of the sensorimotor mask used in our current study.

**Sup-Figure 2**

**
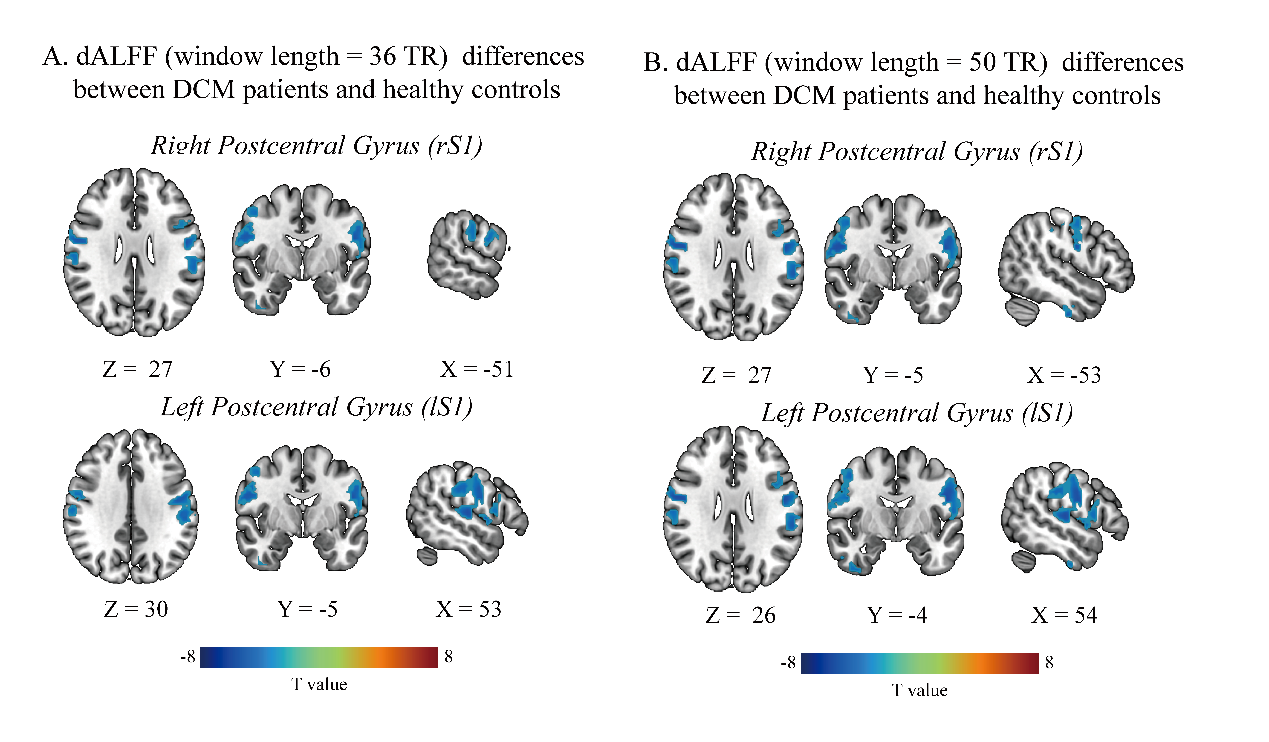
**

The univariate differences in dALFF (36TRs and 50 TRs) between DCM patients and healthy controls. DCM: Degenerative Cervical Myelopathy. DCM: Degenerative Cervical Myelopathy; HC: Healthy Controls; dALFF: dynamic amplitude of low frequency fluctuation

**Sup-Figure 3**


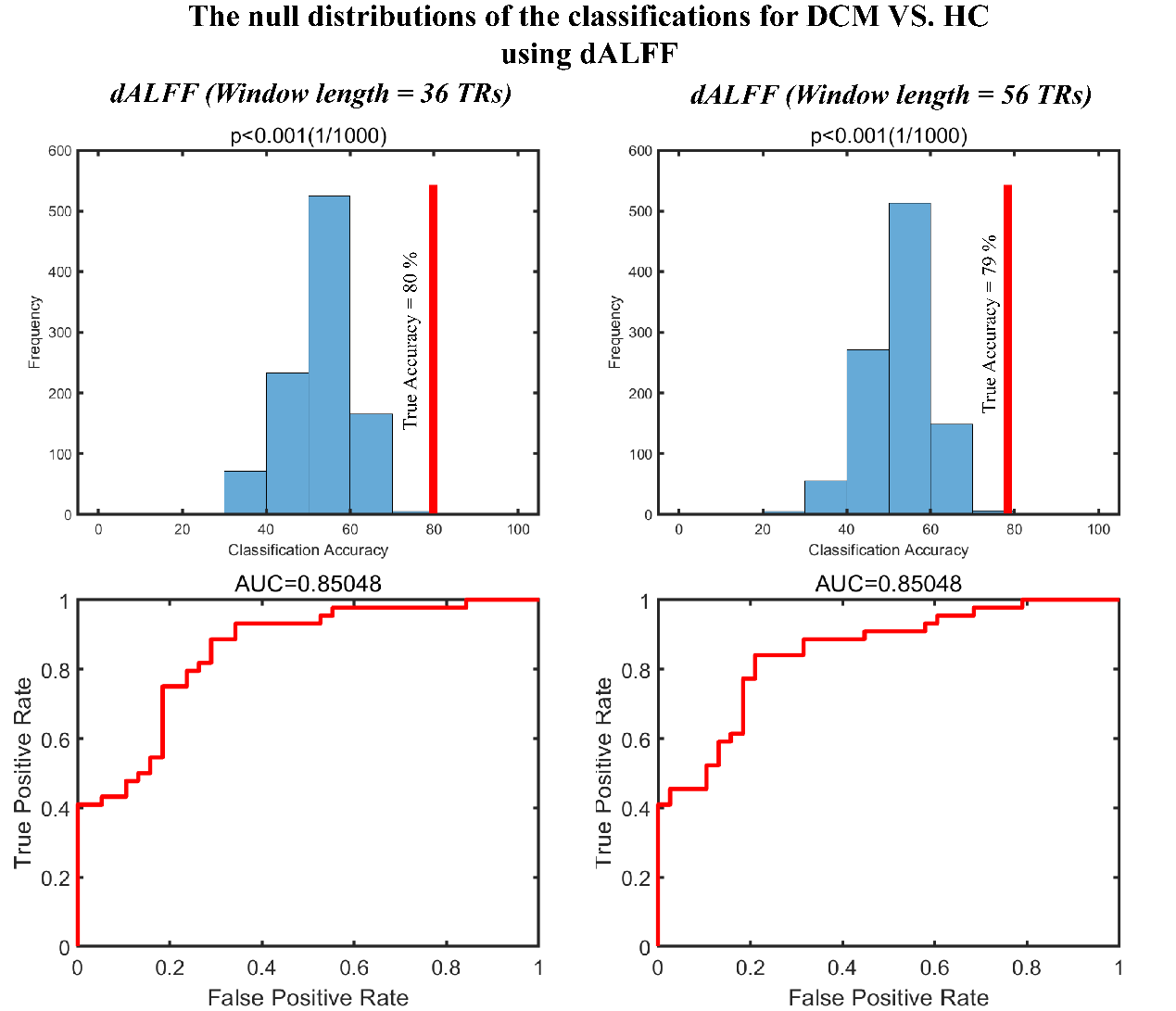


The null distributions of the classifications and receiver operator characteristic curve (ROC curve) for DCM vs. HC using sALFF and dALFF. DCM: Degenerative Cervical Myelopathy; HC: Healthy Controls; sALFF: static amplitude of low frequency fluctuation; dALFF: dynamic amplitude of low frequency fluctuation; AUC: area under the curve.

**Sup-Figure 4**


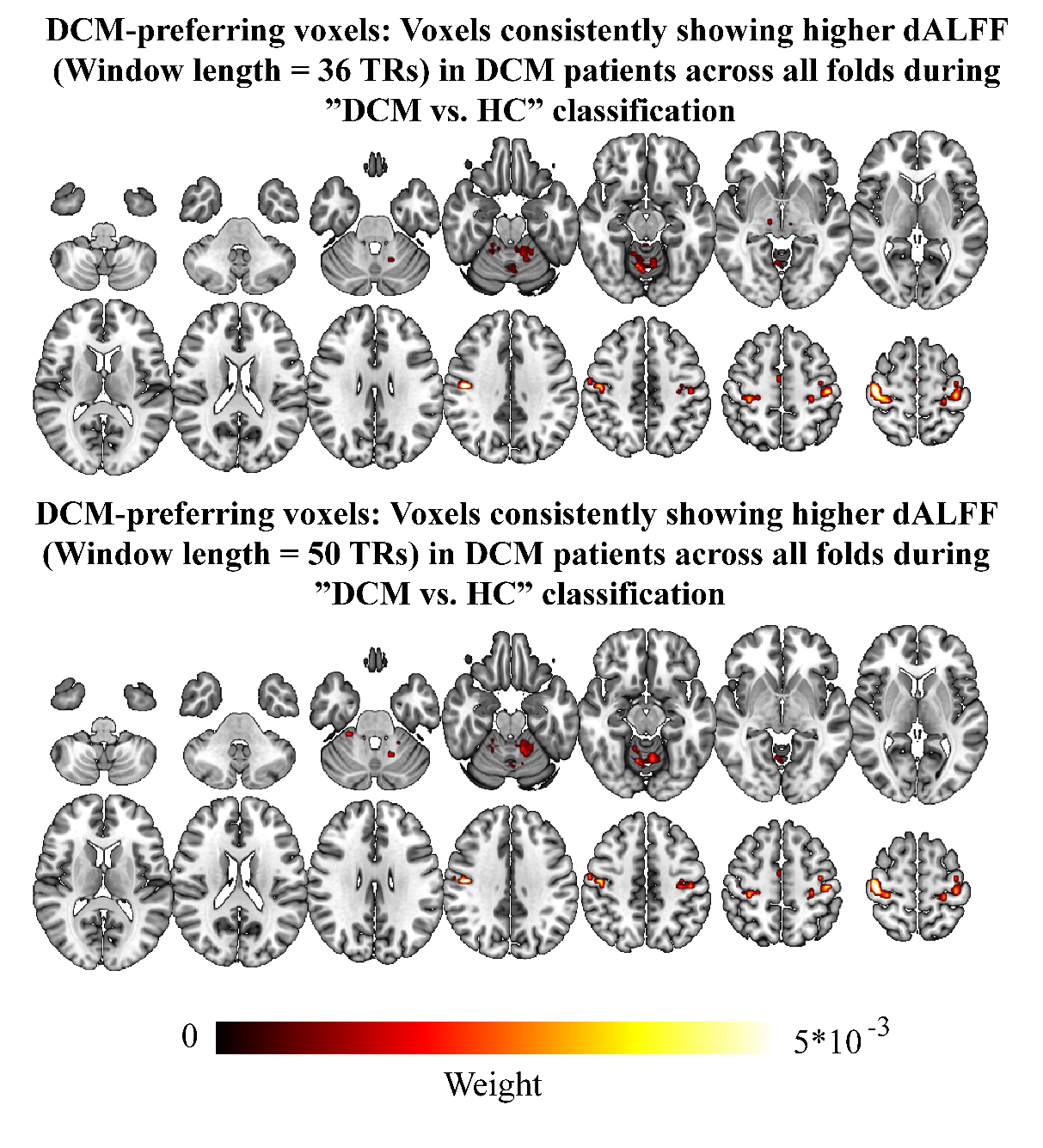


Voxels showed higher weight for classification for “DCM vs. HC” across all folds. Degenerative Cervical Myelopathy; HC: Healthy Controls

**Sup-Figure 5**


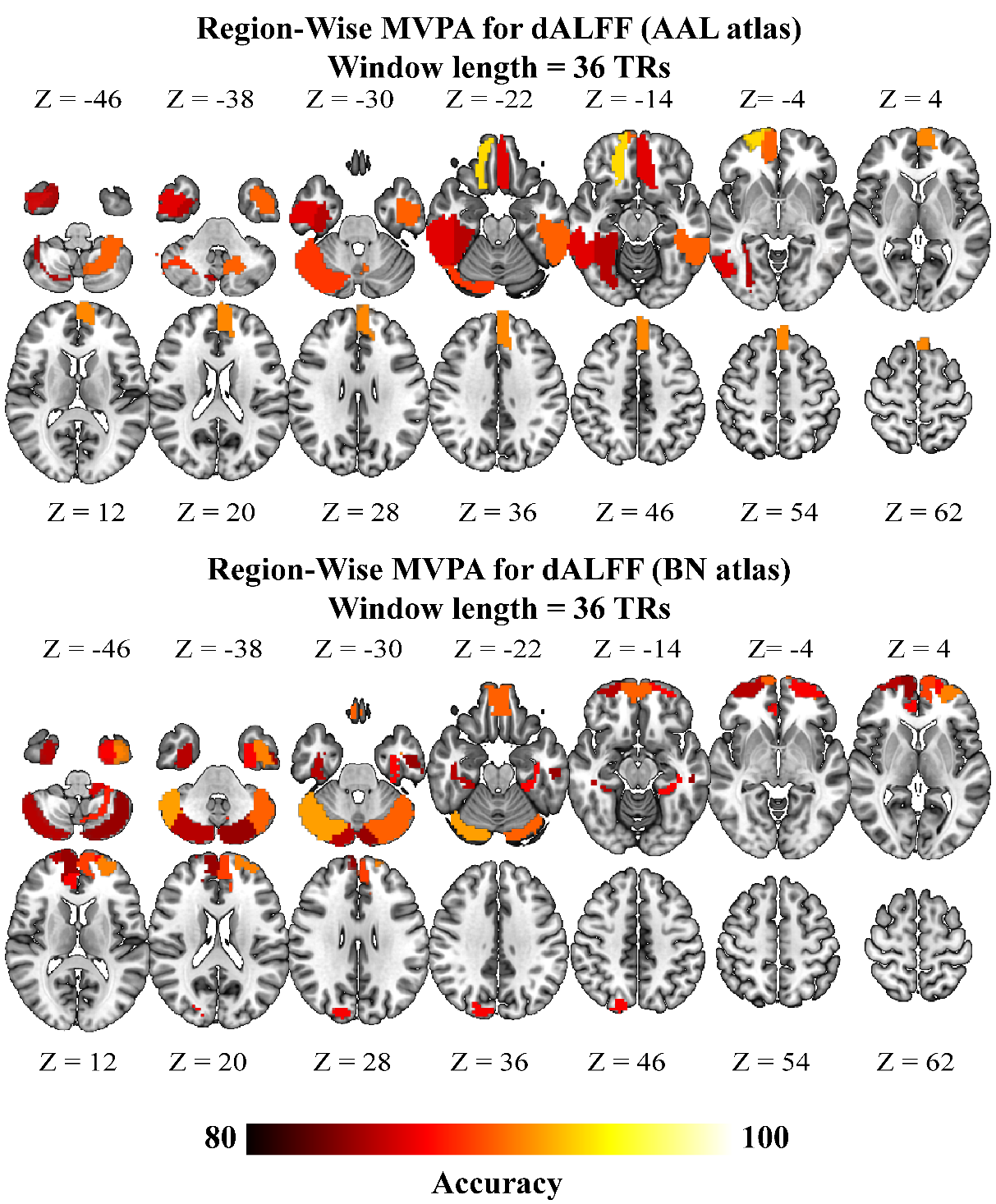


Brain regions with significant classification accuracy for “DCM vs. HC” using static amplitude of low frequency fluctuation (dALFF 36 TRs) (p < 0.05, FWE for multiple comparison correction). DCM: Degenerative Cervical Myelopathy; HC: Healthy Controls; AAL: Anatomical Automatic Labeling; BN: Brainnetome.

**Sup-Figure 6**


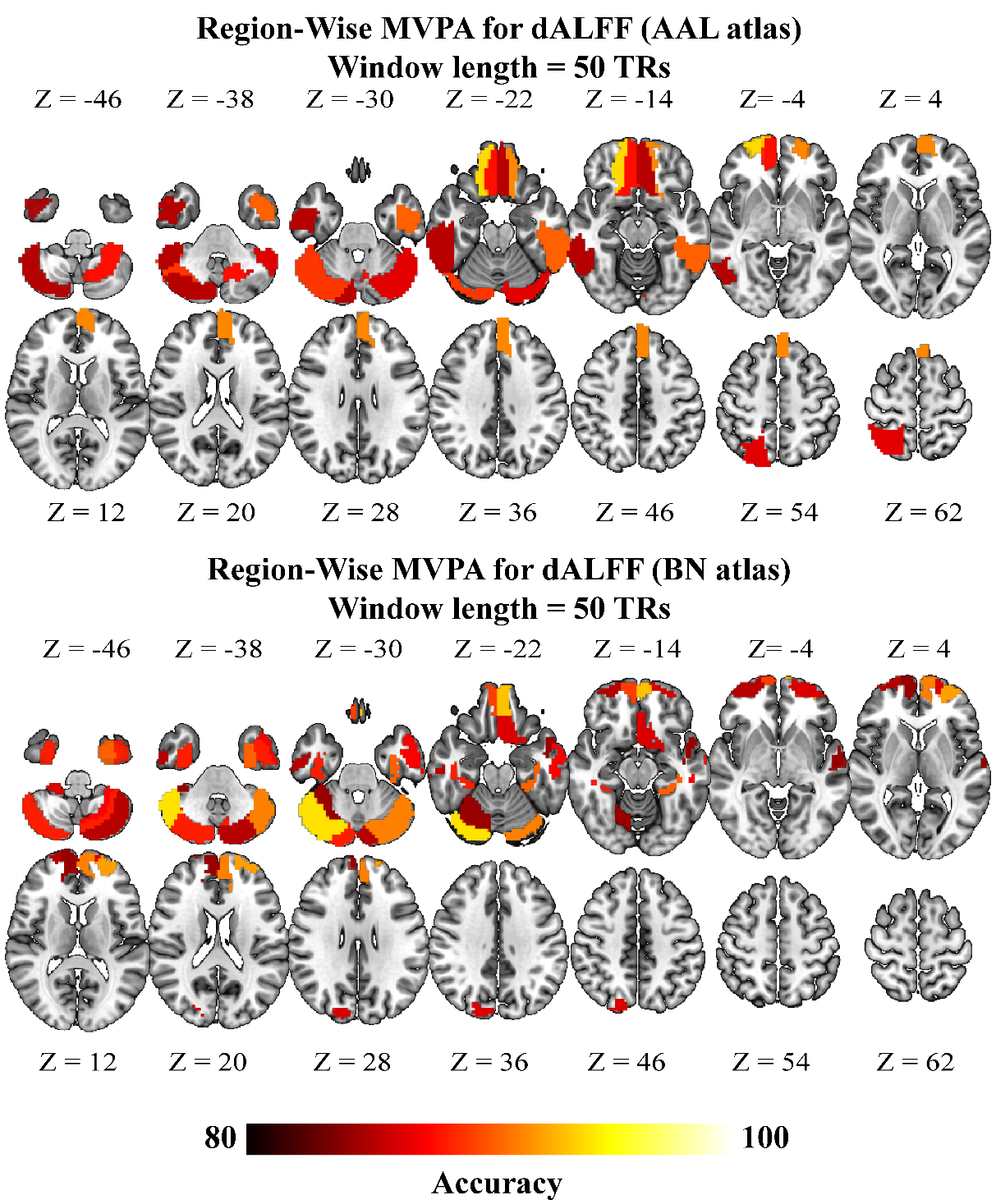


Brain regions with significant classification accuracy for “DCM vs. HC” using static amplitude of low frequency fluctuation (dALFF 50 TRs) (p < 0.05, FWE for multiple comparison correction). DCM: Degenerative Cervical Myelopathy; HC: Healthy Controls; AAL: Anatomical Automatic Labeling; BN: Brainnetome.

**Sup-Figure 7**


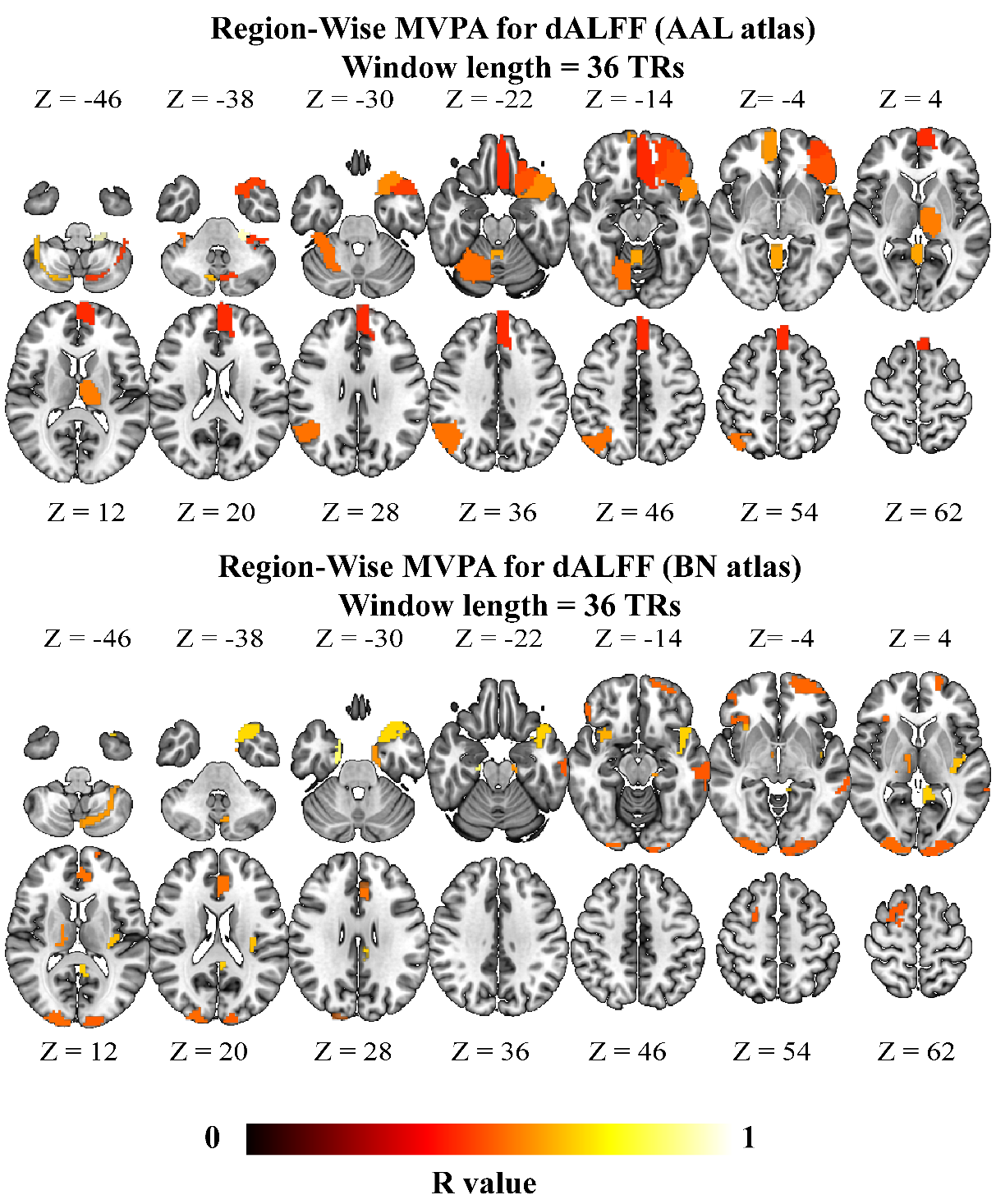


Brain regions with significant correlation coefficients in the prediction of JOA recovery rate using dynamic amplitude of low frequency fluctuation (dALFF 36 TRs) (p < 0.05, FWE for multiple comparison correction). DCM: Degenerative Cervical Myelopathy; HC: Healthy Controls; AAL: Anatomical Automatic Labeling; BN: Brainnetome.

Sup-Figure 8


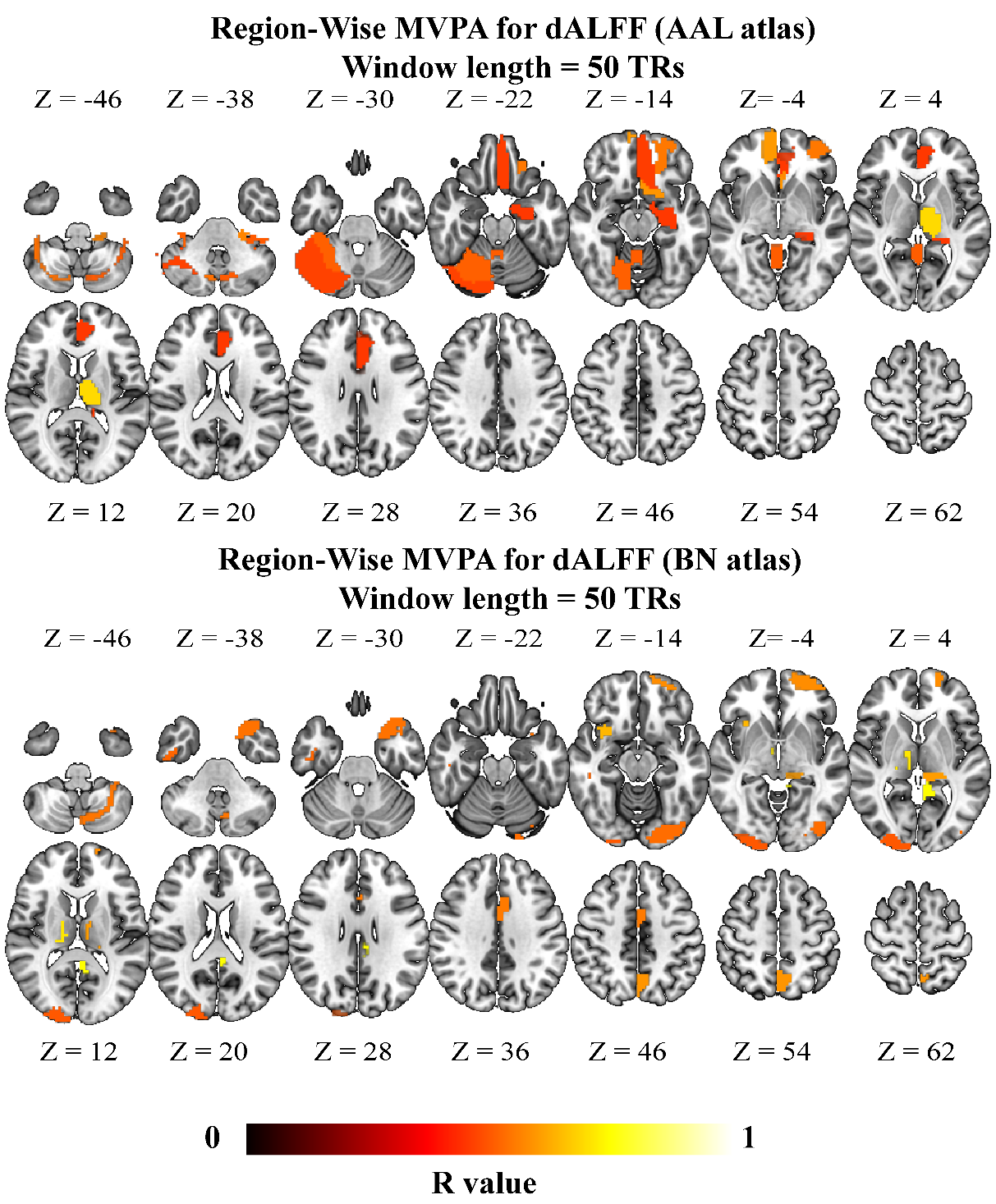


Brain regions with significant correlation coefficients in the prediction of JOA recovery rate using dynamic amplitude of low frequency fluctuation (dALFF 50 TRs) (p < 0.05, FWE for multiple comparison correction). DCM: Degenerative Cervical Myelopathy; HC: Healthy Controls; AAL: Anatomical Automatic Labeling; BN: Brainnetome.

**Sup-Table 1.**

The dALFF and sALFF differences between DCM patients and healthy controls.

| dALFF window length = 36 TRs | | | | | | | | | | |
| --- | --- | --- | --- | --- | --- | --- | --- | --- | --- | --- |
| Brain region | | MNI coordinates | | | | | T value | | Voxel Size | |
| lS1 | | | -54 | 0 | 24 | -6.22 | | | | 435 |
| rS1 | | | 60 | -3 | 27 | -4.83 | | | | 88 |
| rITG | | | 48 | -12 | -42 | -5.31 | | | | 51 |
| lIFO | | | -45 | 12 | 24 | -4.93 | | | | 50 |
| dALFF window length = 50 TRs | | | | | | | | | | |
| lS1 | -51 | | | -6 | 27 | -6.37 | | 501 | | |
| rS1 | 57 | | | -3 | 27 | -6.17 | | 299 | | |
| lITG | -36 | | | -21 | -36 | -6.67 | | 42 | | |
| rITG | 54 | | | -6 | -33 | -6.53 | | 60 | | |

dALFF: dynamic amplitude of low frequency fluctuation; sALFF: static amplitude of low frequency fluctuation; DCM: degenerative cervical myelopathy; lS1: left postcentral gyrus; rS1 right postcentral gyrus; rITG: right inferior temporal gyrus; lIFO: left inferior operculum; rITG: right inferior temporal gyrus.

**Sup-Table 2.**

Brain regions with significant classification accuracy for “DCM vs. HCs” (sALFF)

| dALFF (AAL atlas) |  | dALFF (BN atlas) |  |
| --- | --- | --- | --- |
| Brain region | Accuracy | Brain region | Accuracy |
| Frontal_Sup_L | 90.24 | SFG_L_7_7 | 96.34 |
| Frontal_Sup_Orb_L | 90.24 | SFG_R_7_7 | 90.24 |
| Frontal_Sup_Orb_R | 96.34 | MFG_L_7_3 | 89.02 |
| Olfactory_R | 91.46 | MFG_R_7_7 | 89.02 |
| Frontal_Sup_Medial_L | 91.46 | OrG_L_6_1 | 93.90 |
| Frontal_Mid_Orb_L | 96.34 | OrG_R_6_1 | 90.24 |
| Frontal_Mid_Orb_R | 96.34 | OrG_L_6_4 | 97.56 |
| Rectus_R | 95.12 | OrG_R_6_4 | 92.68 |
| Cingulum_Ant_L | 91.46 | OrG_R_6_5 | 93.90 |
| Cingulum_Ant_R | 93.90 | ITG_L_7_3 | 89.02 |
| Temporal_Mid_L | 91.46 | ITG_R_7_3 | 92.68 |
| Temporal_Mid_R | 91.46 | FuG_L_3_1 | 93.90 |
| Temporal_Inf_R | 96.34 | FuG_R_3_1 | 89.02 |
| Cerebelum_Crus1_R | 91.46 | PhG_L_6_2 | 89.02 |
| Cerebelum_Crus2_R | 98.78 | CG_R_7_2 | 89.02 |
| Cerebelum_8_R | 90.24 | CG_L_7_7 | 92.68 |
|  |  | CG_R_7_7 | 93.90 |
|  |  | BG_L_6_1 | 93.90 |
|  |  | BG_R_6_3 | 90.24 |
|  |  | Cerebelum_Crus1_R | 90.24 |
|  |  | Cerebelum_Crus2_R | 91.46 |
|  |  | Cerebelum_7b_L | 90.24 |
|  |  | Cerebelum_9_L | 93.90 |
|  |  | Cerebelum_8_L | 93.90 |

The brain regions with significant classification accuracy for “DCM vs. HCs” (*p* < 0.05 after FWE correction for multiple comparison correction). sALFF: static amplitude of low frequency fluctuation; DCM: Degenerative Cervical Myelopathy.

**Sup-Table 3.**

Brain regions with significant classification accuracy for “DCM vs. HCs” (dALFF window length = 22 TRs)

| dALFF (AAL atlas) |  | dALFF (BN atlas) |  |
| --- | --- | --- | --- |
| Brain region | Accuracy | Brain region | Accuracy |
| Frontal_Sup_R | 92.68 | SFG_R_7_7 | 92.68 |
| Frontal_Sup_Orb_L | 85.36 | MFG_L_7_7 | 85.36 |
| Frontal_Sup_Orb_R | 95.12 | MFG_R_7_7 | 90.24 |
| Frontal_Sup_Medial_L | 90.24 | OrG_L_6_1 | 89.02 |
| Frontal_Sup_Medial_R | 85.36 | OrG_R_6_1 | 86.58 |
| Frontal_Mid_Orb_R | 86.58 | OrG_L_6_4 | 95.12 |
| Rectus_L | 86.58 | OrG_R_6_4 | 90.24 |
| Rectus_R | 87.80 | OrG_L_6_5 | 85.36 |
| Cingulum_Ant_L | 87.80 | MTG_L_4_2 | 85.36 |
| Temporal_Mid_L | 86.58 | ITG_L_7_1 | 89.02 |
| Temporal_Inf_L | 85.36 | ITG_R_7_1 | 86.58 |
| Temporal_Inf_R | 89.02 | ITG_L_7_7 | 89.02 |
| Cerebelum_Crus1_R | 87.80 | BG_L_6_1 | 92.68 |
| Cerebelum_Crus2_R | 89.02 | BG_L_6_3 | 87.80 |
|  |  | Cerebelum_Crus1_R | 85.36 |

The brain regions with significant classification accuracy for “DCM vs. HCs” (*p* < 0.05 after FWE correction for multiple comparison correction). dALFF: dynamic amplitude of low frequency fluctuation; DCM: Degenerative Cervical Myelopathy.

**Sup-Table 4.**

Brain regions with significant classification accuracy for “DCM vs. HCs” (dALFF window length = 36 TRs)

| dALFF (AAL atlas) |  | dALFF (BN atlas) |  |
| --- | --- | --- | --- |
| Brain region | Accuracy | Brain region | Accuracy |
| Frontal_Sup_Orb_R | 93.9% | SFG_L_7_7 | 86.6% |
| Frontal_Sup_Medial_L | 91.5% | SFG_R_7_7 | 80.5% |
| Frontal_Mid_Orb_R | 90.3% | MFG_L_7_3 | 89.1% |
| Rectus_L | 86.6% | MFG_L_7_7 | 84.2% |
| Fusiform_R | 85.4% | MFG_R_7_7 | 81.7% |
| Temporal_Inf_L | 90.2% | OrG_L_6_4 | 87.8% |
| Temporal_Inf_R | 86.6% | OrG_ R_6_4 | 87.8% |
| Cerebelum_Crus1_R | 89.1% | ITG_L_7_3 | 89.1% |
| Cerebelum_7b_R | 85.3% | ITG_L_7_4 | 80.5% |
| Cerebelum_8_L | 90.2% | FuG_L_3_1 | 84.1% |
|  |  | FuG_R_3_1 | 84.2% |
|  |  | CG_ R_7_7 | 87.8% |
|  |  | LOcC _ R_2_1 | 90.3% |
|  |  | Cerebelum_Crus1_L | 80.5% |
|  |  | Cerebelum_Crus1_R | 81.7% |
|  |  | Cerebelum_Crus2_R | 85.4% |
|  |  | Cerebelum_Crus2_L | 85.9% |

The brain regions with significant classification accuracy for “DCM vs. HCs” (*p* < 0.05 after FWE correction for multiple comparison correction). dALFF: dynamic amplitude of low frequency fluctuation; DCM: Degenerative Cervical Myelopathy.

**Sup-Table 5.**

Brain regions with significant classification accuracy for “DCM vs. HCs” (dALFF window length = 50 TRs)

| dALFF (AAL atlas) |  | dALFF (BN atlas) |  |
| --- | --- | --- | --- |
| Brain region | Accuracy | Brain region | Accuracy |
| Frontal_Sup_Orb_L | 91.5% | SFG_L(R)_7_7 | 89.1 |
| Frontal_Sup_Orb_R | 93.6% | SFG_L(R)_7_7 | 80.5 |
| Frontal_Sup_Medial_L | 91.5% | MFG_L_7_3 | 90.3 |
| Frontal_Mid_Orb_R | 87.8% | MFG_L_7_7 | 82.9 |
| Rectus_L | 85.4% | MFG_ R_7_7 | 81.7 |
| Rectus_L | 87.8% | OrG_L_6_4 | 91.5 |
| Parietal_Sup_R | 86.6% | OrG_ R_6_4 | 86.6 |
| Temporal_Inf_L | 90.3% | OrG_L_6_5 | 82.9 |
| Temporal_Inf_R | 85.4% | STG_L_6_6 | 80.8 |
| Cerebelum_Crus1_L | 86.6% | MTG_L_4_2 | 84.1 |
| Cerebelum_Crus1_R | 89.1% | ITG_L_7_3 | 82.7 |
| Cerebelum_Crus2_R | 85.4% | ITG_L_7_4 | 87.8 |
| Cerebelum_7b_R | 85.5% | ITG_ R_7_4 | 85.3 |
| Cerebelum_8_L | 87.8% | FuG_L_3_1 | 82.9 |
|  |  | FuG_ R_3_1 | 80.5 |
|  |  | LOcC _ R_2_1 | 89.1 |
|  |  | Cerebelum_Crus1_L | 92.7 |
|  |  | Cerebelum_Crus1_R | 81.7 |
|  |  | Cerebelum_Crus2_R | 85.4 |
|  |  | Cerebelum_7b_L | 85.6 |
|  |  | Cerebelum_7b_R | 84.1 |
|  |  | Cerebelum_8_L | 82.1 |
|  |  | Cerebelum_8_R | 80.6 |

The brain regions with significant classification accuracy for “DCM vs. HCs” (*p* < 0.05 after FWE correction for multiple comparison correction). dALFF: dynamic amplitude of low frequency fluctuation; DCM: Degenerative Cervical Myelopathy.

**Sup-Table 6.**

The brain regions with significant R values in the prediction of JOA recovery rate in DCM patients using sALFF.

| sALFF (AAL atlas) |  | sALFF (BN atlas) |  |
| --- | --- | --- | --- |
| Brain region | R value | Brain region | R value |
| Frontal_Sup_L | 0.52 | MFG_ R_7_6 | 0.47 |
| Frontal_Mid_L | 0.43 | MFG_L_7_7 | 0.64 |
| Frontal_Mid_R | 0.42 | IFG_ R_6_6 | 0.45 |
| Frontal_Inf_Tri_R | 0.60 | PhG_L_6_6 | 0.42 |
| Rolandic_Oper_R | 0.53 | PoG_L_4_1 | 0.43 |
| Supp_Motor_Area_L | 0.43 | PoG_R_4_2 | 0.44 |
| Cingulum_Mid_L | 0.43 | LOcC _ R_4_3 | 0.48 |
| Cingulum_Mid_R | 0.42 | LOcC_L_4_4 | 0.50 |
| Hippocampus_L | 0.55 | Cerebelum_Crus1_L | 0.53 |
| Cuneus_R | 0.41 | Cerebelum_Crus1_R | 0.45 |
| Occipital_Sup_R | 0.54 | Cerebelum_Crus2_R | 0.52 |
| Occipital_Sup_L | 0.47 |  |  |
| Fusiform_R | 0.41 |  |  |
| Paracentral_Lobule_L | 0.49 |  |  |
| Temporal_Sup_L | 0.45 |  |  |
| Temporal_Pole_Mid_L | 0.43 |  |  |
| Cerebelum_Crus1_L | 0.42 |  |  |
| Cerebelum_Crus1_R | 0.51 |  |  |
| Cerebelum_Crus2_R | 0.53 |  |  |
| Cerebelum_7b_R | 0.51 |  |  |
| Cerebelum_8_L | 0.51 |  |  |

The brain regions with significant correlation coefficients for predicting JOA recovery rate (*p* < 0.05 after FWE correction for multiple comparison correction). sALFF: static amplitude of low frequency fluctuation; dALFF: dynamic amplitude of low frequency fluctuation; DCM: Degenerative Cervical Myelopathy.

**Sup-Table 7.**

The brain regions with significant R values in the prediction of JOA recovery rate in DCM patients using sALFF. (dALFF window length = 22 TRs)

| sALFF |  | sALFF |  |
| --- | --- | --- | --- |
| Brain region | R value | Brain region | R value |
| Frontal_Sup_Orb_R | 0.47 | SFG_R_7_4 | 0.50 |
| Frontal_Mid_R | 0.47 | SFG_L_7_7 | 0.48 |
| Rolandic_Oper_R | 0.46 | MFG_L(R)_7_7 | 0.47 |
| Frontal_Sup_Medial_L | 0.45 | CG_L_7_3 | 0.45 |
| Frontal_Mid_Orb_R | 0.45 | CG_R_7_7 | 0.56 |
| Insula_L | 0.45 | LOcC _R_4_3 | 0.57 |
| Cingulum_Ant_L | 0.50 | LOcC_L_4_4 | 0.49 |
| Cingulum_Ant_R | 0.49 | Amyg_R_2_1 | 0.52 |
| ParaHippocampal_R | 0.47 | Amyg_L_2_2 | 0.48 |
| Cuneus_L | 0.46 | BG_R_6_2 | 0.50 |
| Lingual_L | 0.43 |  | 0.51 |
| Occipital_Mid_R | 0.44 |  | 0.47 |
| Occipital_Inf_L | 0.45 |  | 0.52 |
| Occipital_Inf_R | 0.46 |  |  |
| Heschl_R | 0.49 |  |  |
| Temporal_Pole_Sup_R | 0.45 |  |  |
| Temporal_Pole_Mid_L | 0.51 |  |  |
| Cerebelum_Crus1_L | 0.50 |  |  |
| Cerebelum_Crus1_R | 0.48 |  |  |
| Cerebelum_6_R | 0.48 |  |  |
| Cerebelum_7b_R | 0.47 |  |  |
| Vermis_8 | 0.47 |  |  |

The brain regions with significant correlation coefficients for predicting JOA recovery rate (*p* < 0.05 after FWE correction for multiple comparison correction). sALFF: static amplitude of low frequency fluctuation; dALFF: dynamic amplitude of low frequency fluctuation; DCM: Degenerative Cervical Myelopathy.

**Sup-Table 8.**

The brain regions with significant R values in the prediction of JOA recovery rate in DCM patients using sALFF. (dALFF window length = 36 TRs)

| sALFF |  | sALFF |  |
| --- | --- | --- | --- |
| Brain region | R value | Brain region | R value |
| Frontal_Mid_Orb_L | 0.47 | SFG_R_7_4 | 0.40 |
| Frontal_Inf_Orb_L | 0.49 | MFG_L_7_7 | 0.41 |
| Frontal_Sup_Medial_L | 0.45 | OrG_ R_6_6 | 0.41 |
| Frontal_Mid_Orb_R | 0.51 | STG_L_6_1 | 0.55 |
| Rectus_L | 0.45 | STG_L_6_5 | 0.54 |
| Angular_R | 0.47 | MTG_L_4_1 | 0.40 |
| Thalamus_L | 0.48 | PhG_L_6_4 | 0.47 |
| Temporal_Pole_Sup_L | 0.50 | PhG_R_6_4 | 0.68 |
| Temporal_Pole_Mid_L | 0.47 | INS_L_6_1 | 0.52 |
| Cerebelum_6_R | 0.47 | INS_R_6_2 | 0.50 |
| Cerebelum_7b_L | 0.46 | CG_L_7_3 | 0.43 |
| Cerebelum_7b_R | 0.45 | CG_L_7_4 | 0.54 |
| Cerebelum_10_L | 0.65 | LOcC _L_4_3 | 0.40 |
| Vermis_4_5 | 0.48 | LOcC _R_4_3 | 0.42 |
|  |  | Tha_R_8_8 | 0.45 |
|  |  | Cerebelum_7b_L | 0.48 |

The brain regions with significant correlation coefficients for predicting JOA recovery rate (*p* < 0.05 after FWE correction for multiple comparison correction). sALFF: static amplitude of low frequency fluctuation; dALFF: dynamic amplitude of low frequency fluctuation; DCM: Degenerative Cervical Myelopathy.

**Sup-Table 9.**

The brain regions with significant R values in the prediction of JOA recovery rate in DCM patients using sALFF. (dALFF window length = 50 TRs)

| sALFF |  | sALFF |  |
| --- | --- | --- | --- |
| Brain region | R value | Brain region | R value |
| Frontal_Mid_Orb_L | 0.48 | MFG_L_7_7 | 0.46 |
| Olfactory_L | 0.51 | STG_L_6_1 | 0.48 |
| Frontal_Mid_Orb_R | 0.51 | ITG_R_7_1 | 0.46 |
| Rectus_L | 0.45 | PCun_L_4_1 | 0.47 |
| Cingulum_Ant_L | 0.46 | INS_ R_6_2 | 0.51 |
| Thalamus_L | 0.46 | CG_L_7_4 | 0.59 |
| Cerebelum_Crus1_R | 0.58 | CG_L_7_5 | 0.49 |
| Cerebelum_6_R | 0.47 | LOcC _ R_4_3 | 0.45 |
| Cerebelum_7b_L | 0.45 | LOcC_L_4_4 | 0.47 |
| Cerebelum_7b_R | 0.47 | Tha_L_8_6 | 0.48 |
| Vermis_4_5 | 0.49 | Tha_ R_8_8 | 0.57 |
| Cerebelum_10_L | 0.45 | Cerebelum_7b_R | 0.48 |

The brain regions with significant correlation coefficients for predicting JOA recovery rate (*p* < 0.05 after FWE correction for multiple comparison correction). sALFF: static amplitude of low frequency fluctuation; dALFF: dynamic amplitude of low frequency fluctuation; DCM: Degenerative Cervical Myelopathy.
